# Supplementary material for: Design, Synthesis and Preliminary Biological Evaluation of Benzylsulfone Coumarin Derivatives as Anti-Cancer Agents
Source: Molecules. 2019 Nov 7;24(22):4034. doi: 10.3390/molecules24224034 (PMC6891324; doi:10.3390/molecules24224034)

Design, Synthesis and Preliminary Biological Evaluation of Novel Benzylsulfone Coumarin Derivatives as Anticancer Agents

Tao Wang[1,2], Tao Peng[2], Xiaoxue Wen[2], Gang Wang[2], Yunbo Sun[2], Shuchen Liu[2,*], Shouguo Zhang[2,*], and Lin Wang[1,2,*]

[1] College of Life Science and Bio-engineering, Beijing University of Technology, Beijing 100124, P.R. China

[2] Beijing Institute of Radiation Medicine, Beijing 100850, P.R. China

Supplementary Materials

1. Synthesis of Intermediates

1.1. 5-nitrosalicyladehyde (**4b**)

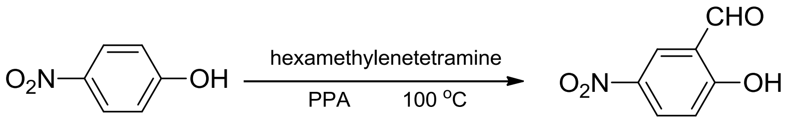

A mixture of 4-nitrophenol (7.5 g, 54 mmol, 1.0 eq.), hexamethylene tetramine (7.5 g, 54 mmol, 1.0 eq.), and 75% PPA (60 mL) was agitated for 5 hours at 100 ℃. Subsequently, the mixture was placed in 100 mL of ethyl acetate and 200 mL of water, and agitated until the mixture became completely dissolved. In addition, as a result of additionally adding 100 ml of ethyl acetate to the foregoing solution, the solution separated into 2 phases. The water phase was removed and the remaining solution was washed with saturated brine (50 mL*2), then the organic layer was dried with anhydrous Na_2SO_4, filtered, and concentrated under vacuum to obtain the desired product **4b** as a yellow solid, yield, 33.0%; m.p. 127-129 ^oC. ^1H NMR (400 MHz, DMSO-d_6) δ: 12.22 (s, 1H, -CHO), 10.30 (s, 1H, -OH), 8.43 (d, 1H, J = 2.8 Hz, H-6), 8.35 (d, 1H, J = 8.8 Hz, H-4), 7.19 (d, 1H, J = 8.8 Hz, H-3).

1.2. 5-Bromosalicylaldehyde (**4c**)

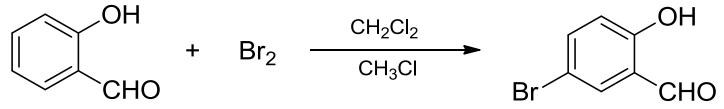

To a solution of salicylaldehyde (3.66 g, 30 mmol, 1.0 eq.) in 20 mL dichloromethane, 5.28 g (33 mmol, 1.1 eq.) bromine in 60 mL chloroform was added dropwisely over a period of 4-5 h. After completion of the reaction (TLC monitoring), the solvent was evaporated to obtain the crude product, which was recrystallized in 90% ethanol to yield the desired product. Yield, 64.2%; white solid, m.p. 105-107 ^oC. ^1H NMR (400 MHz, $CDCl_3$) δ: 10.94 (s, 1H, -CHO); 9.84 (s, 1H, -OH); 7.68 (d, 1H, J = 2.4 Hz, H-6); 7.61 (d, 1H, J = 8.8 Hz, H-4); 6.92 (d, 1H, J = 8.8 Hz, H-3).

1.3. General Procedure for the Preparation of Benzylthioacetic Acids (2a-2j).

The following benzylthioacetic acids were synthesized according to the procedure described in the literature and identified by comparing their melting points and properties with the literature data.

2-(2-Fluorobenzylthio)acetic Acid (**2a**). Condensation of 2-fluorobenzyl chloride **1a** with mercaptoacetic acid yielded the corresponding 2-(2-fluorobenzylthio)acetic

acid. The yield of this reaction was 98.5%, giving a pale yellow oil. It was identified by comparing its physical data with the literature data [1].

2-(3-Fluorobenzylthio)acetic Acid (**2b**). Condensation of 3-fluorobenzyl chloride **1b** with mercaptoacetic acid yielded the corresponding 2-(3-fluorobenzylthio)acetic acid. The yield of this reaction was 98.1%, giving a pale yellow oil. It was identified by comparing its physical data with the literature data [1].

2-(4-Fluorobenzylthio)acetic Acid (**2c**). Condensation of 4-fluorobenzyl chloride **1c** with mercaptoacetic acid yielded the corresponding 2-(4-fluorobenzylthio)acetic acid. The yield of this reaction was 94.8%, giving a pale yellow oil. It was identified by comparing its physical data with the literature data [2].

2-(2-Chlorobenzylthio)acetic Acid (**2d**). Condensation of 2-chlorobenzyl chloride **1d** with mercaptoacetic acid yielded the corresponding 2-(2-chlorobenzylthio)acetic acid. The yield of this reaction was 89.8%, giving a white solid with a melting point of 52-53 ^{o}C. It was identified by comparing its physical data with the literature data [3].

2-(2-Bromobenzylthio)acetic Acid (**2e**). Condensation of 2-bromobenzyl bromide **1e** with mercaptoacetic acid yielded the corresponding 2-(2-bromobenzylthio)acetic acid. The yield of this reaction was 94.6%, giving a white solid with a melting point of 49-51 ^{o}C. It was identified by comparing its physical data with the literature data [3].

2-(3-Bromobenzylthio)acetic Acid (**2f**). Condensation of 3-bromobenzyl bromide **1f** with mercaptoacetic acid yielded the corresponding 2-(3-bromobenzylthio)acetic acid. The yield of this reaction was 91.7%, giving a pale yellow solid with a melting point of 87-89 ^{o}C. It was identified by comparing its physical data with the literature data [4].

2-(4-Bromobenzylthio)acetic Acid (**2g**). Condensation of 4-bromobenzyl bromide **1g** with mercaptoacetic acid yielded the corresponding 2-(4-bromobenzylthio)acetic acid. The yield of this reaction was 91.2%, giving a pale yellow solid with a melting point of 73-76 ^{o}C. It was identified by comparing its physical data with the literature data [4].

2-(3-Methylbenzylthio)acetic Acid (**2h**). Condensation of 3-methylbenzyl chloride **1h** with mercaptoacetic acid yielded the corresponding 2-(3-methylbenzylthio)acetic acid. The yield of this reaction was 91.5%, giving a pale yellow solid with a melting point of 72-74 ^{o}C. It was identified by comparing its physical data with the literature data [5].

2-(4-Methylbenzylthio)acetic Acid (**2i**). Condensation of 4-methylbenzyl chloride **1i** with mercaptoacetic acid yielded the corresponding 2-(4-methylbenzylthio)acetic acid. The yield of this reaction was 78.7%, giving a pale yellow solid with a melting point of 65-67 ^{o}C. It was identified by comparing its physical data with the literature data [6].

2-[2-(Trifluoromethyl)benzylthio]acetic Acid (**2j**). Condensation of 2-(trifluoro methyl)benzyl bromide **1j** with mercaptoacetic acid yielded the corresponding 2-[2-(trifluoromethyl)benzylthio]acetic acid. The yield of this reaction was 94.5%, giving a pale yellow oil. It was identified by comparing its physical data with the

literature data [5].

1.4. General Procedure for the Preparation of Benzylsulfonylacetic Acids (3a-3j).

The following benzylsulfonylacetic acids were synthesized according to the procedure described in the literature and identified by comparing their melting points and properties with the literature data.

2-[(2-Fluorobenzyl)sulfonyl]acetic Acid (**3a**). Oxidation of 2-(2-fluorobenzylthio)acetic acid **2a** with 30% hydrogen peroxide yielded the corresponding 2-[(2-fluorobenzyl)sulfonyl]acetic acid. The yield of this reaction was 58.9%, giving a white solid with a melting point of 134.5-136 ^{o}C. It was identified by comparing its physical data with the literature data [7].

2-[(3-Fluorobenzyl)sulfonyl]acetic Acid (**3b**). Oxidation of 2-(3-fluorobenzylthio)acetic acid **2b** with 30% hydrogen peroxide yielded the corresponding 2-[(3-fluorobenzyl)sulfonyl]acetic acid. The yield of this reaction was 83.3%, giving a white solid with a melting point of 126-128 ^{o}C. It was identified by comparing its physical data with the literature data [7].

2-[(4-Fluorobenzyl)sulfonyl]acetic Acid (**3c**). Oxidation of 2-(4-fluorobenzylthio)acetic acid **2c** with 30% hydrogen peroxide yielded the corresponding 2-[(4-fluorobenzyl)sulfonyl]acetic acid. The yield of this reaction was 60.5%, giving a white solid with a melting point of 160-164 ^{o}C. It was identified by comparing its physical data with the literature data [2].

2-[(2-Chlorobenzyl)sulfonyl]acetic Acid (**3d**). Oxidation of 2-(2-chlorobenzylthio)acetic acid **2d** with 30% hydrogen peroxide yielded the corresponding 2-[(2-chlorobenzyl)sulfonyl]acetic acid. The yield of this reaction was 70.0%, giving a white solid with a melting point of 162-165°C. It was identified by comparing its physical data with the literature data [8].

2-[(2-Bromobenzyl)sulfonyl]acetic Acid (**3e**). Oxidation of 2-(2-bromobenzylthio)acetic acid **2e** with 30% hydrogen peroxide yielded the corresponding 2-[(2-bromobenzyl)sulfonyl]acetic acid. The yield of this reaction was 78.7%, giving a white solid with a melting point of 175-177 ^{o}C. It was identified by comparing its physical data with the literature data [3].

2-[(3-Bromobenzyl)sulfonyl]acetic Acid (**3f**). Oxidation of 2-(3-bromobenzylthio)acetic acid **2f** with 30% hydrogen peroxide yielded the corresponding 2-[(3-bromobenzyl)sulfonyl]acetic acid. The yield of this reaction was 85.8%, giving a white solid with a melting point of 179-181 ^{o}C. It was identified by comparing its physical data with the literature data [3].

2-[(4-Bromobenzyl)sulfonyl]acetic Acid (**3g**). Oxidation of 2-(4-bromobenzylthio)acetic acid **2g** with 30% hydrogen peroxide yielded the corresponding 2-[(4-bromobenzyl)sulfonyl]acetic acid. The yield of this reaction was 87.7%, giving a white solid with a melting point of 177-179 ^{o}C. It was identified by comparing its physical data with the literature data [9].

2-[(3-Methylbenzyl)sulfonyl]acetic Acid (**3h**). Oxidation of 2-(3-methylbenzylthio)acetic acid **2h** with 30% hydrogen peroxide yielded the corresponding 2-[(3-methylbenzyl)sulfonyl]acetic acid. The yield of this reaction was

82.8%, giving a white solid with a melting point of 134-136 °C. It was identified by comparing its physical data with the literature data [5].

2-[(4-Methylbenzyl)sulfonyl]acetic Acid (**3i**). Oxidation of 2-(4-methylbenzylthio)acetic acid **2i** with 30% hydrogen peroxide yielded the corresponding 2-[(4-methylbenzyl)sulfonyl]acetic acid. The yield of this reaction was 43.2%, giving a white solid with a melting point of 145-146 °C. It was identified by comparing its physical data with the literature data [5].

2-{[2-(Trifluoromethyl)benzyl]sulfonyl}acetic Acid (**3j**). Oxidation of 2-[2-(trifluoromethyl)benzylthio]acetic acid **2j** with 30% hydrogen peroxide yielded the corresponding 2-{[2-(trifluoromethyl)benzyl]sulfonyl}acetic acid. The yield of this reaction was 79.0%, giving a white solid with a melting point of 168-169 °C. It was identified by comparing its physical data with the literature data [8].

References

[1] Kojima, K; Koyama, K; Kurata, H; Fukumi, H; Tabata, K; Yasuda, H. Preparation of disubstituted thiophenes and ulcer inhibitors. *Jpn. Kokai Tokkyo Koho*, **1996**, JP 08127581, A 19960521.

[2] Zhou, N.; Feng, T.; Shen, X.; Cui, J.; Wu, R.; Wang, L.; Chen, H. Synthesis, characterization, and radioprotective activity of α,β-unsaturated aryl sulfone analogs and their Tempol conjugates. *MedChemComm*. **2017**, *8*, 1063-1068.

[3] Janczewski, M.; Ksiezopolski, J. Effect of molecular structure on optical properties of sulfoxide systerms, m-Bromobenzylsulfinylacetic acids and some if their derivatives, Part VII. *Pol. J. Chem.* **1984**, *58*, 103-106.

[4] Abraham, D.J.; Kennedy, P.E.; Mehanna, A.S.; Patwa, D.C.; Williams, F.L. Design, synthesis, and testing of potential antisickling agents. 4. structure activity relationships of benzyloxy and phenoxy acids. *J. Med. Chem*. **1984**, *16*, 967-981.

[5] Janczewski, M.; Janowski, W. Effect of molecular structure on optical properties of sulfoxide systems. Optical relations in the group of isomeric o-, m-, and p-methylbenzylsulfinylacetic acids. *Roczniki Chemii*. **1975**, *49*, 1961-1962.

[6] Mccaw, P.G.; Buckley N.M.; Eccles K.S.; Lawrence S.E.; Maguire A.R.; Collins S.G. Synthesis of Cyclic α-Diazo-β-keto Sulfoxides in Batch and Continuous Flow. *J. Org. Chem.* **2017**, *82*, 3666-3679.

[7] Bordwell, F.G.; Wolfinger, M.D.; O'Dwyer, J.B. Synthesis of dihalomethyl and alpha-haloalkyl sulfones by the halogenative decarboxylation of alpha-aryl- and alpha-alkylsulfonylalkane carboxylic acids. *ChemInform*. 1974, *5*, 2516-2519.

[8] Ning X.L.; Guo Y.; Wang X.W.; Ma X.Y.; Tian C.; Shi X.Q.; Zhu R.Z.; Cheng C.; Du Y.S.; Ma Z.Z.; et al. Design, synthesis, and biological evaluation of (E)-3,4-dihydroxystyryl aralkyl sulfones and sulfoxides as novel multifunctional neuroprotective Agents. *J. Med. Chem*. **2014**, *57*, 4302-4312.

[9] Janczewski, M.; Ksiezopolski, J. Effect of molecular structure on optical properties of sulfoxide systerms, p-Bromobenzylsulfinylacetic acids and some if their derivatives, Part III. *Pol. J. Chem*. **1981**, *55*, 535-546.

2. Copies of ^{1}H-NMR and ^{13} C-NMR for target compounds (5a -5o)

^{1}H-NMR of Compound **5a**:

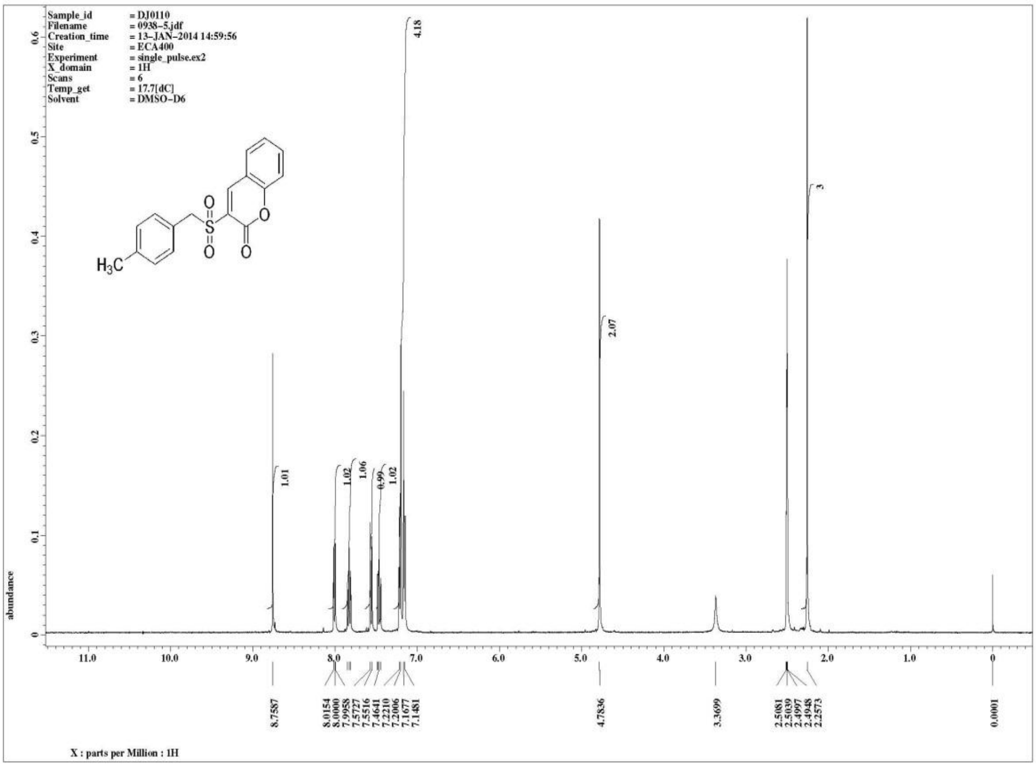

^{13}C-NMR of Compound **5a**:

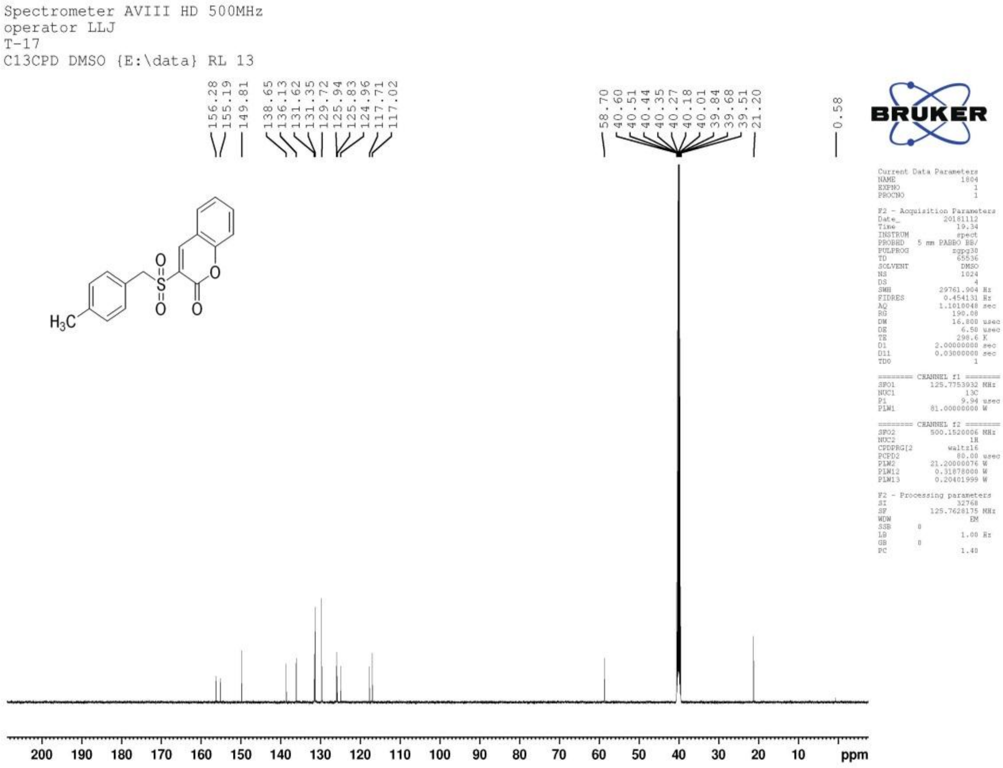

^{1}H-NMR of Compound **5b**:

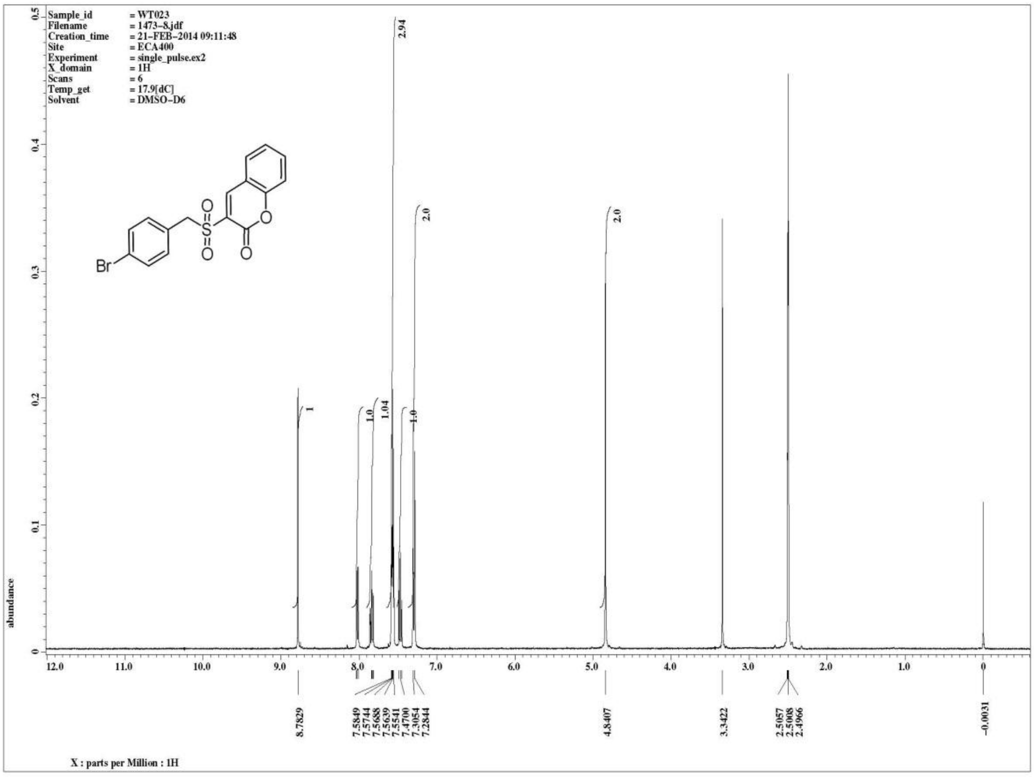

^{13}C-NMR of Compound **5b**:

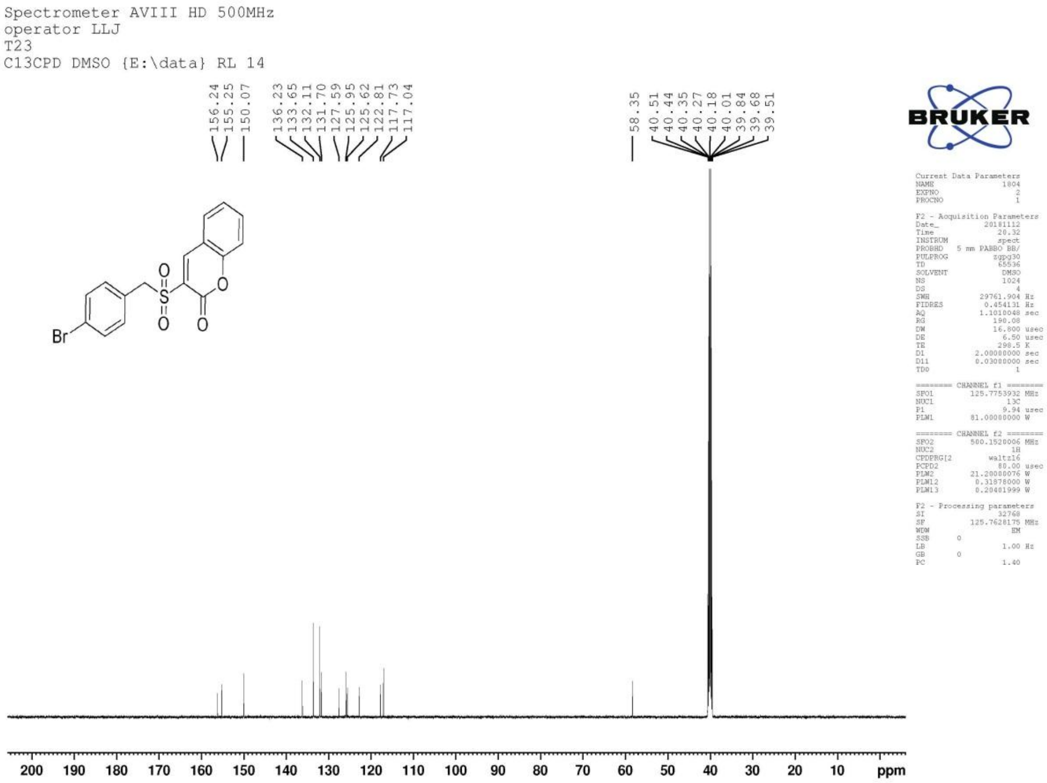

^{1}H-NMR of Compound **5c**:

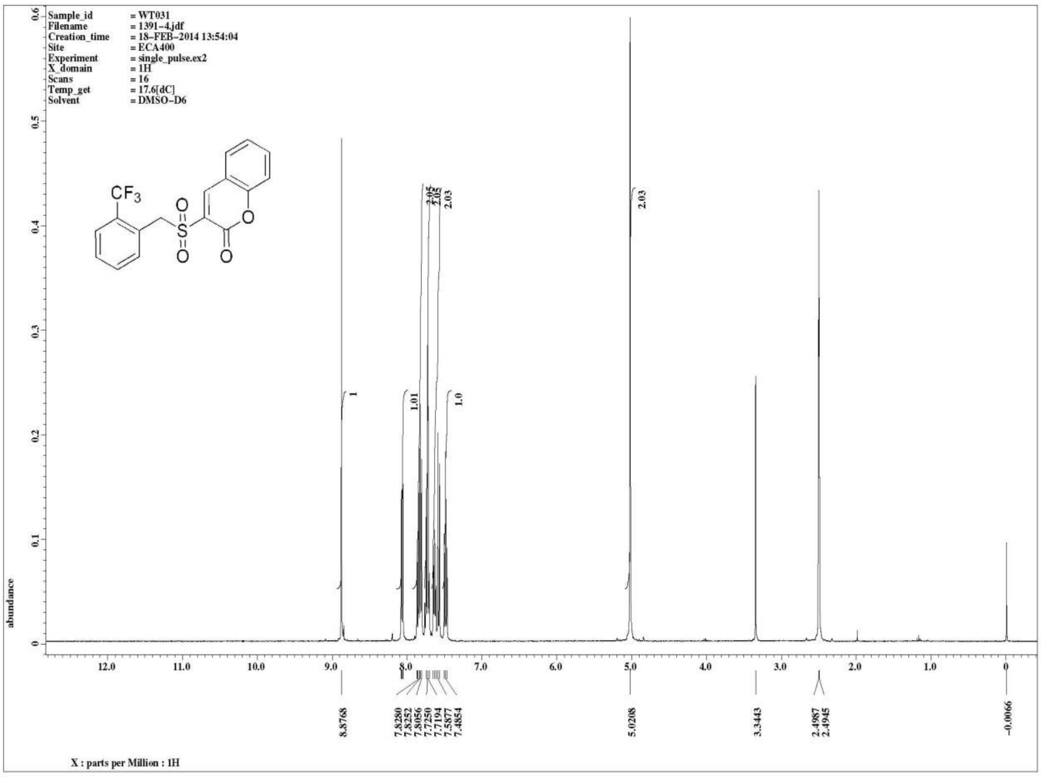

^{13}C-NMR of Compound **5c**:

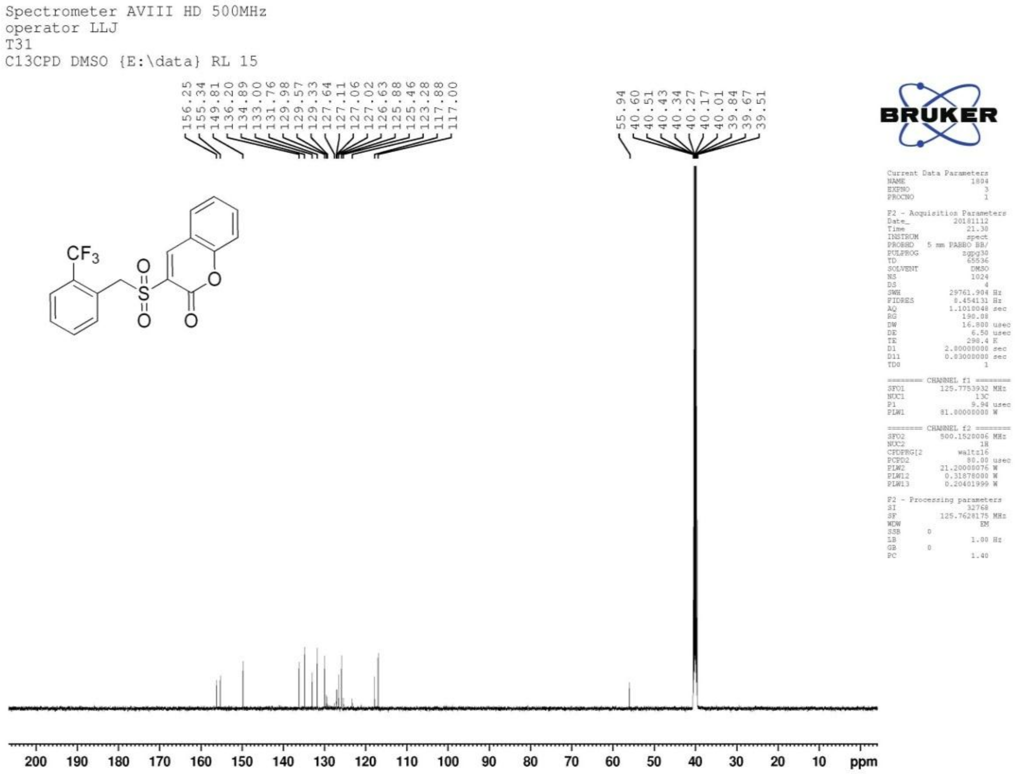

^{1}H-NMR of Compound **5d**:

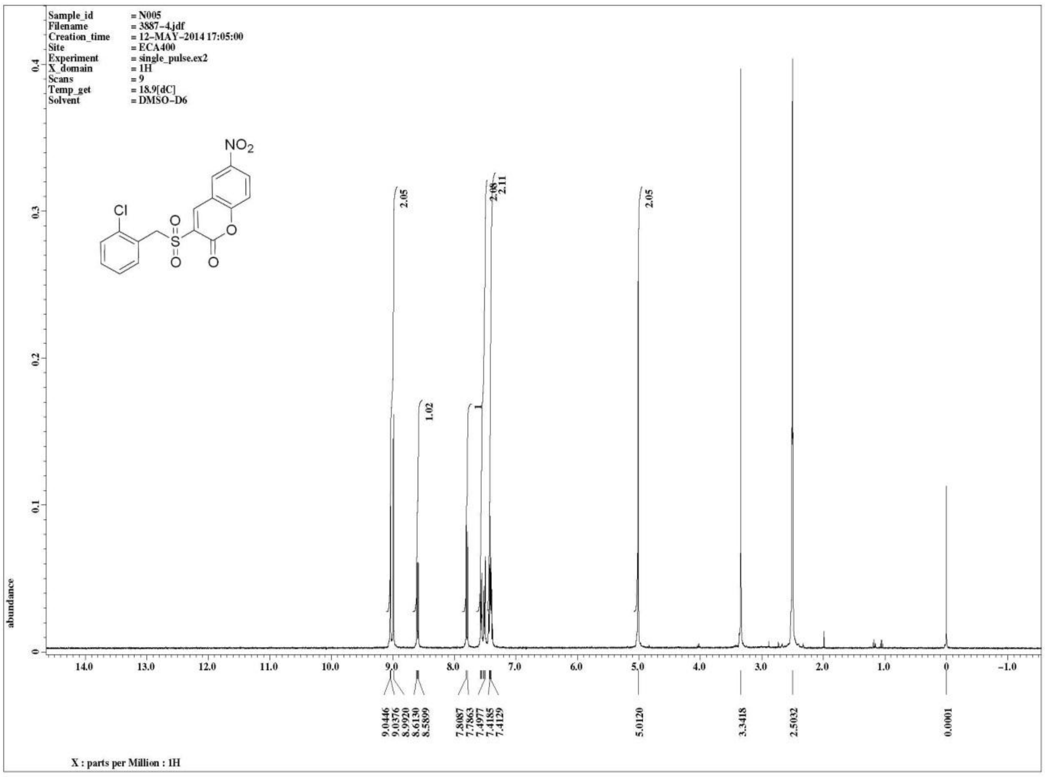

^{13}C-NMR of Compound **5d**:

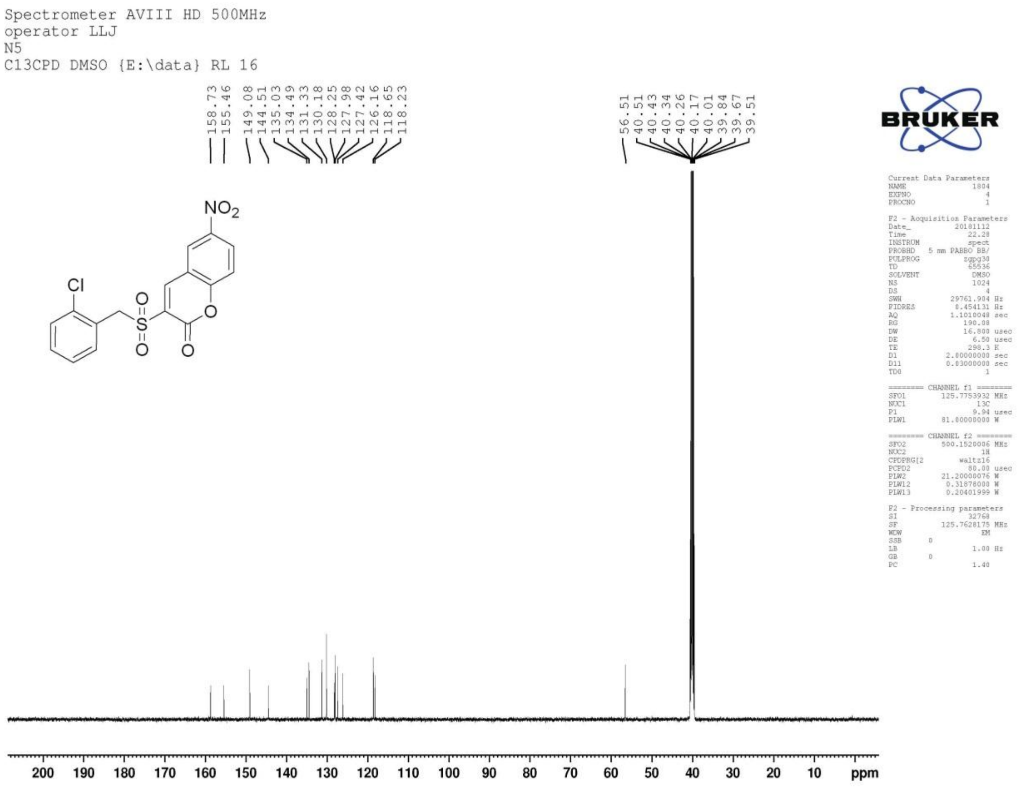

^{1}H-NMR of Compound **5e**:

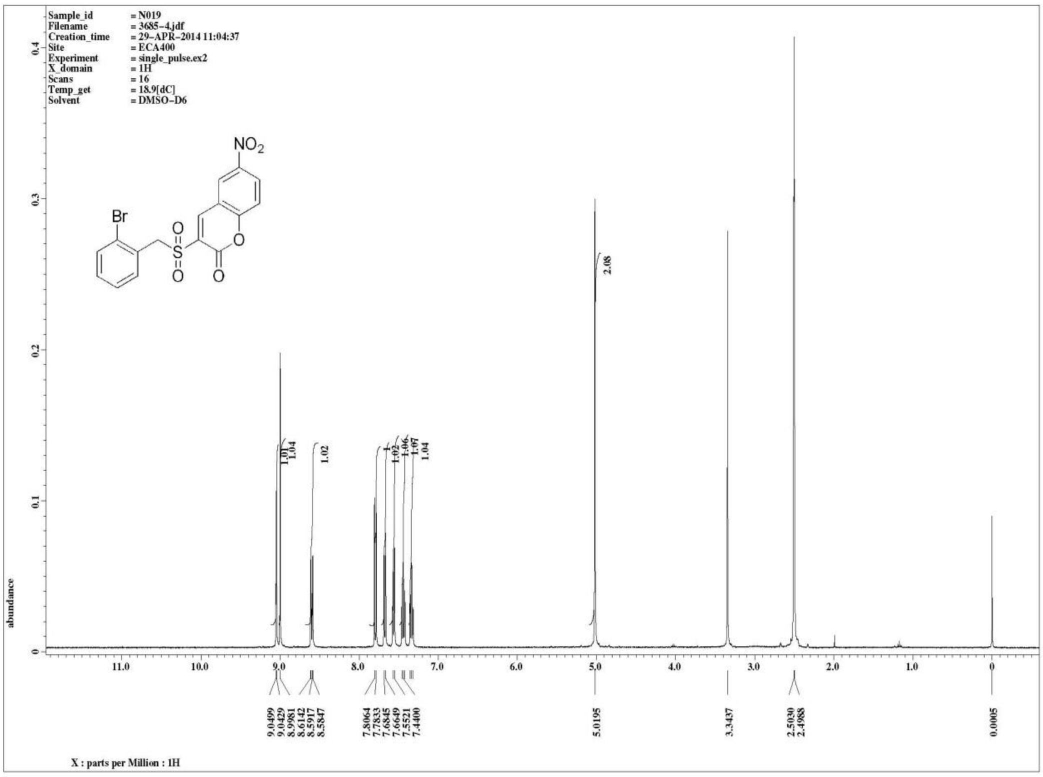

^{13}C-NMR of Compound **5e**:

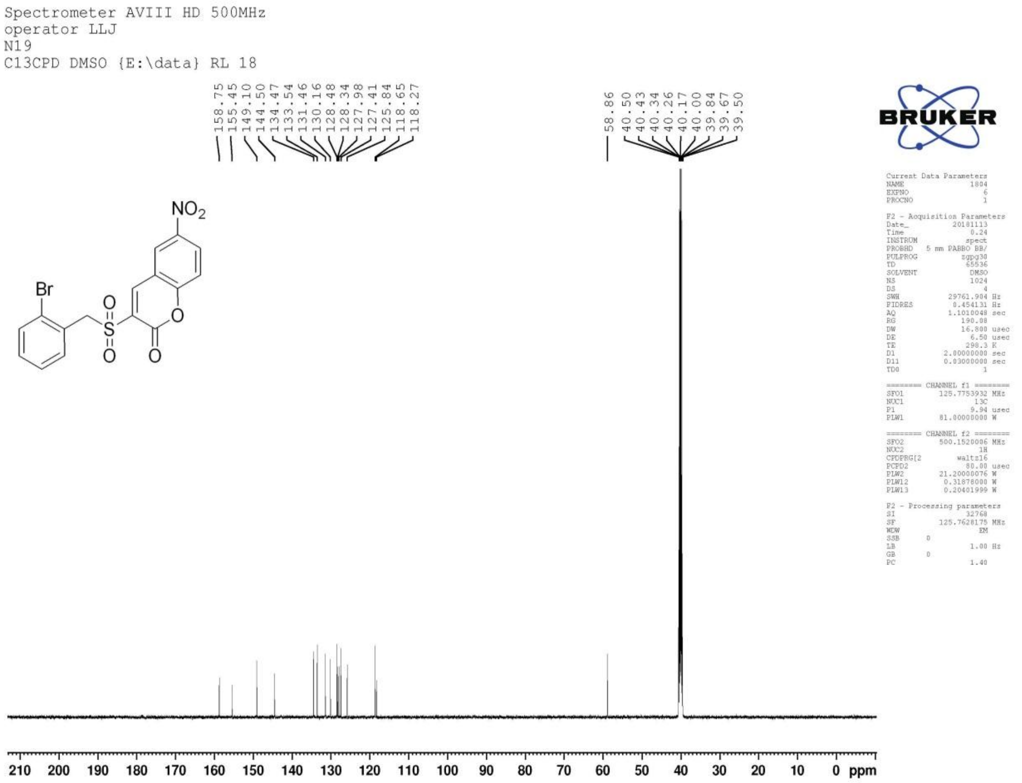

^{1}H-NMR of Compound **5f**:

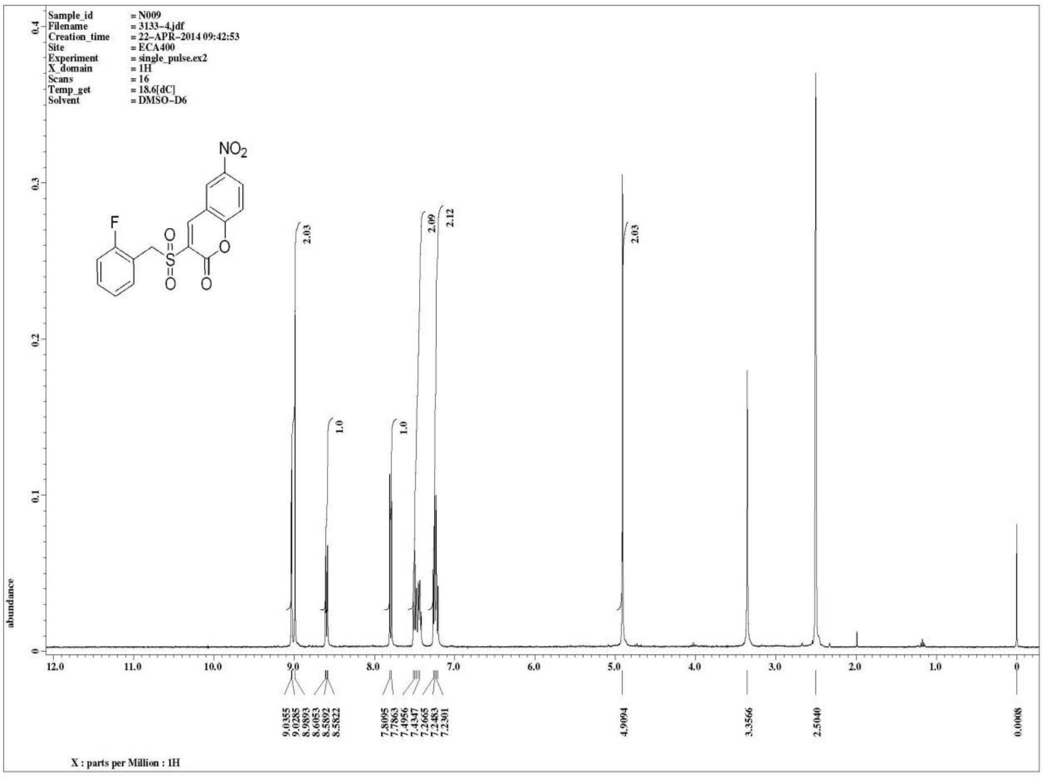

^{13}C-NMR of Compound **5f**:

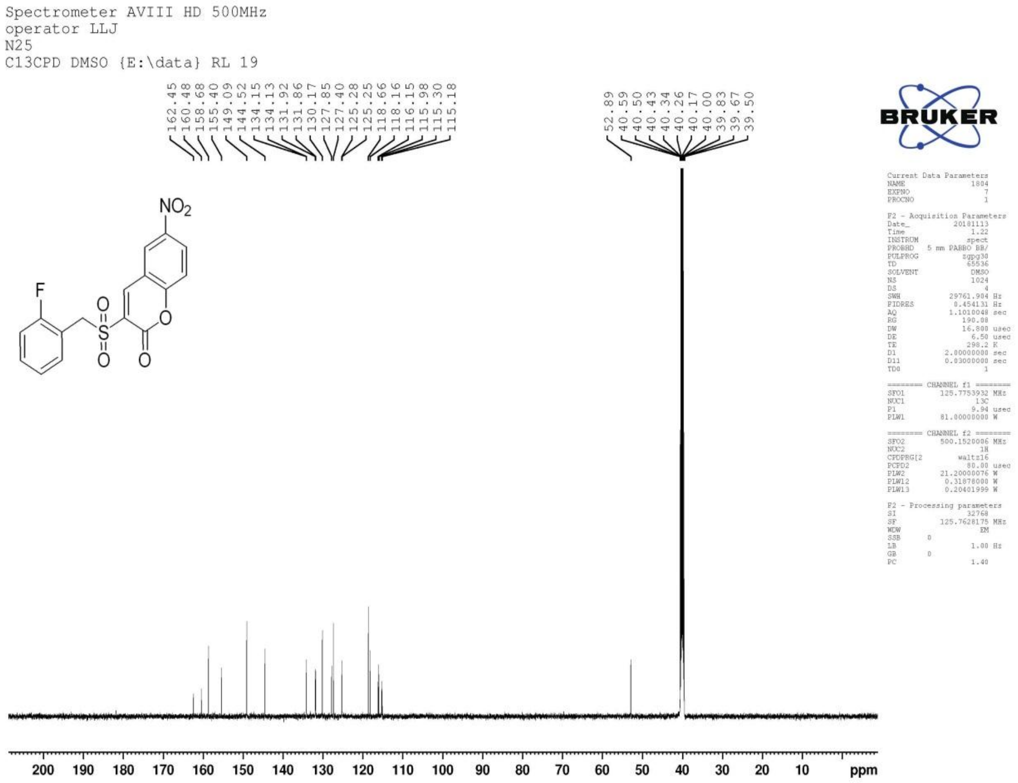

^{1}H-NMR of Compound **5g**:

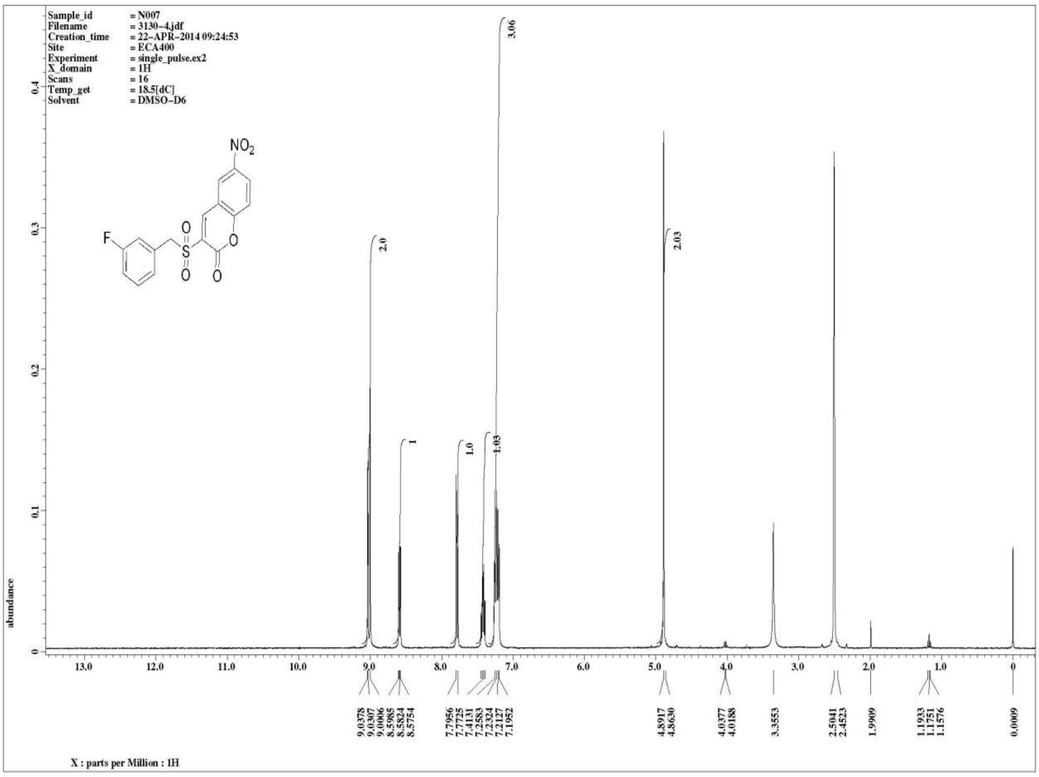

^{13}C-NMR of Compound **5g**:

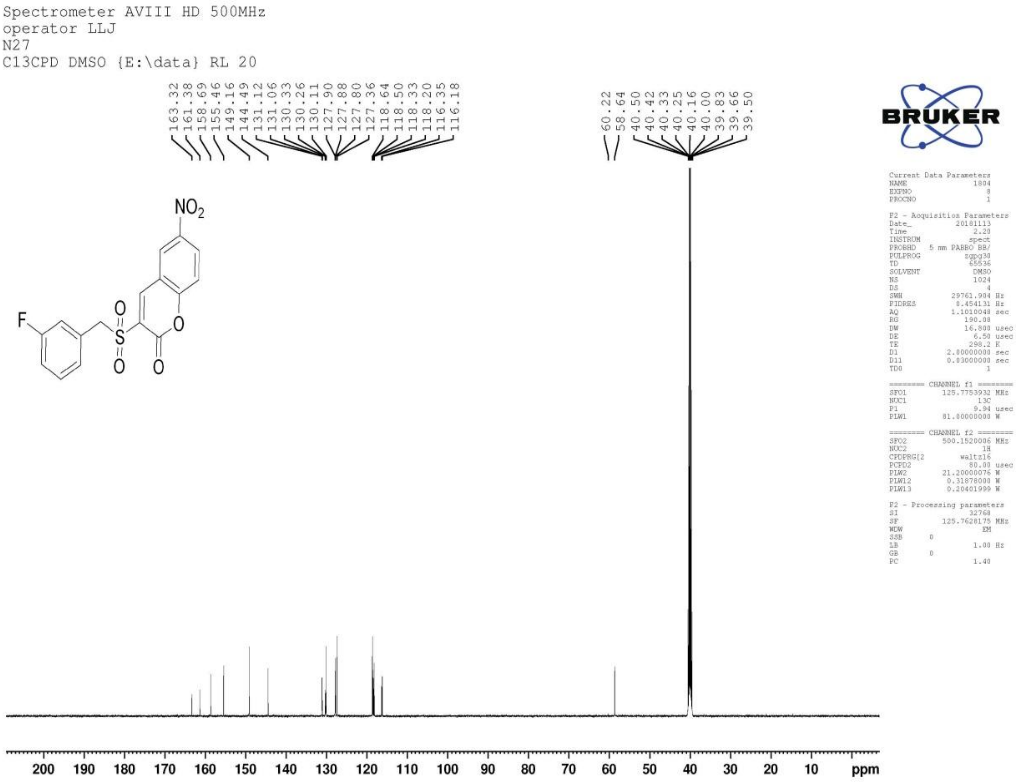

^{1}H-NMR of Compound **5h**:

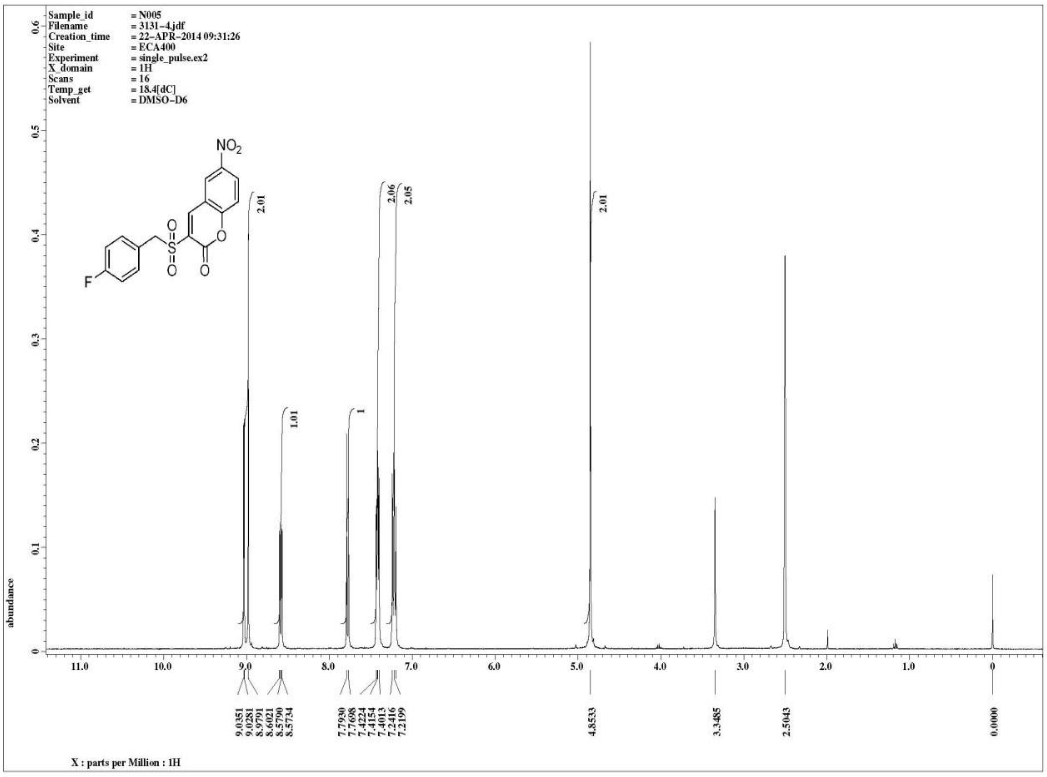

^{13}C-NMR of Compound **5h**:

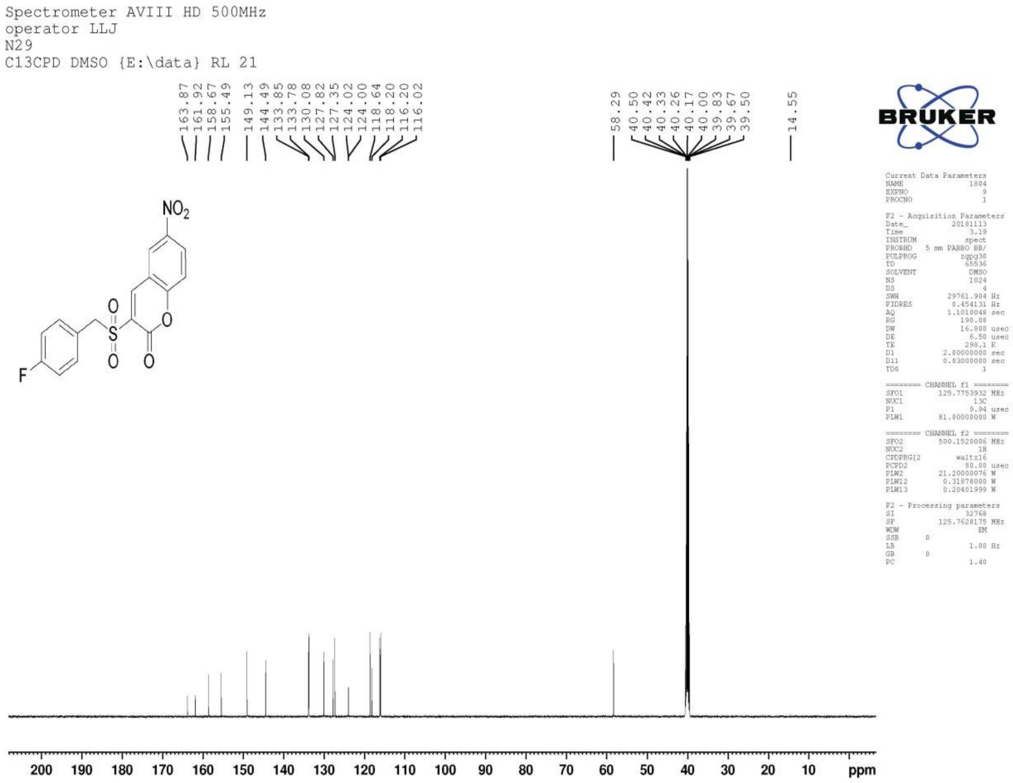

^{1}H-NMR of Compound **5i**:

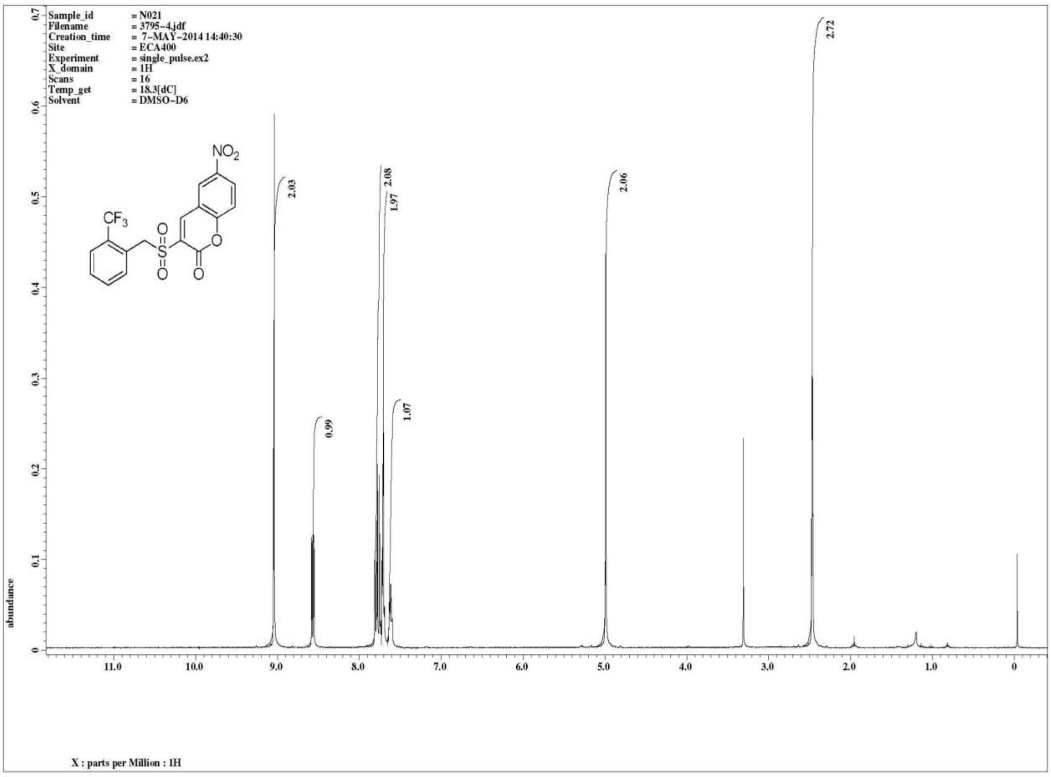

^{13}C-NMR of Compound **5i**:

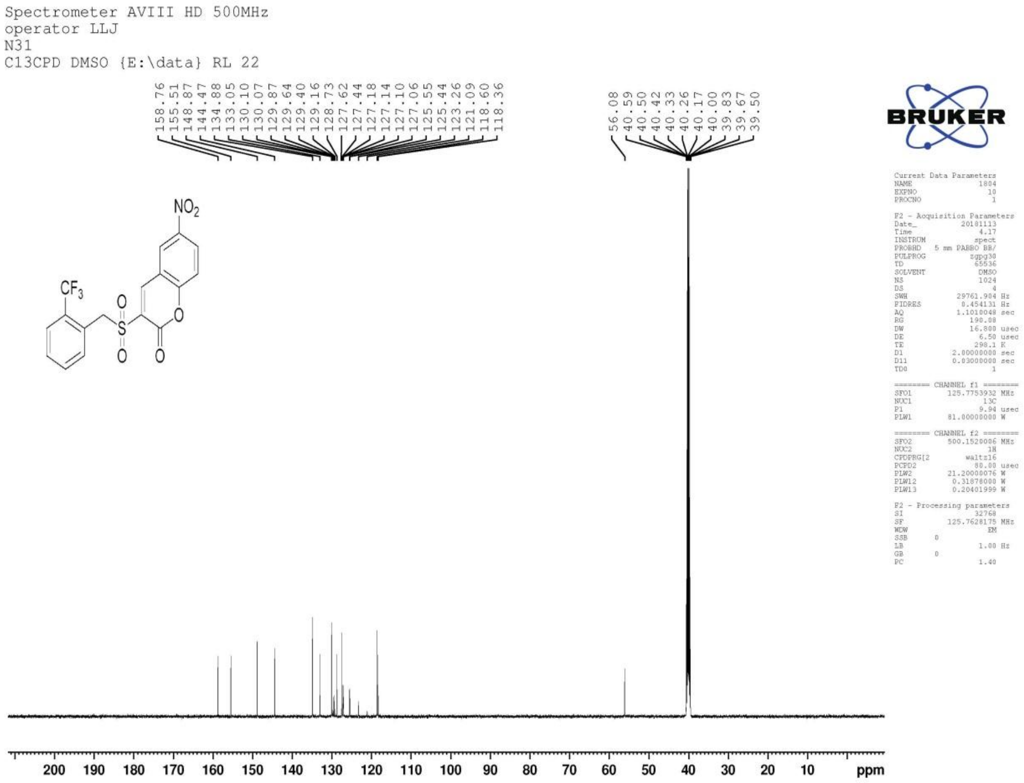

^{1}H-NMR of Compound **5j**:

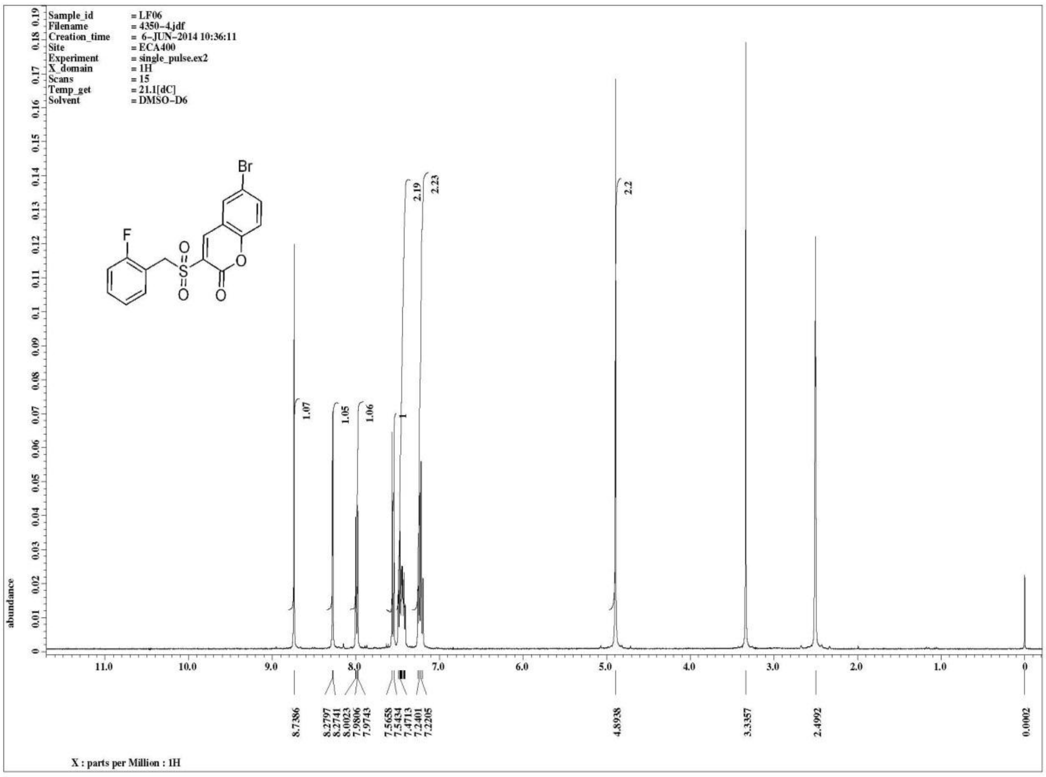

^{13}C-NMR of Compound **5j**:

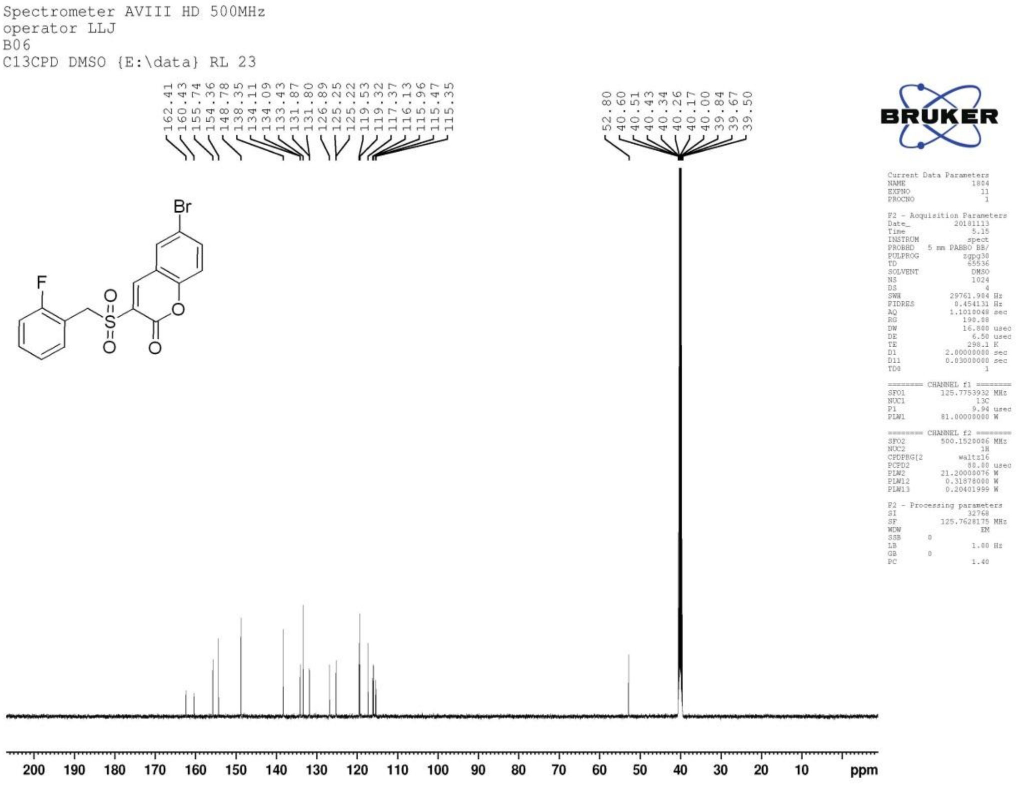

^{1}H-NMR of Compound **5k**:

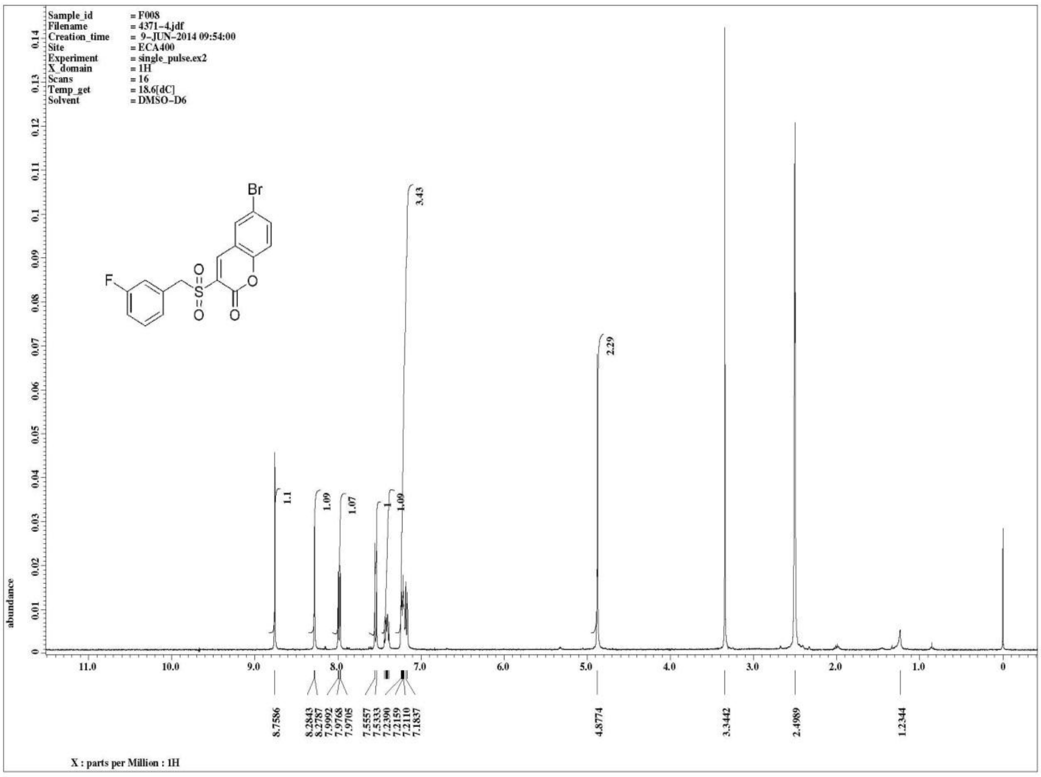

^{13}C-NMR of Compound **5k**:

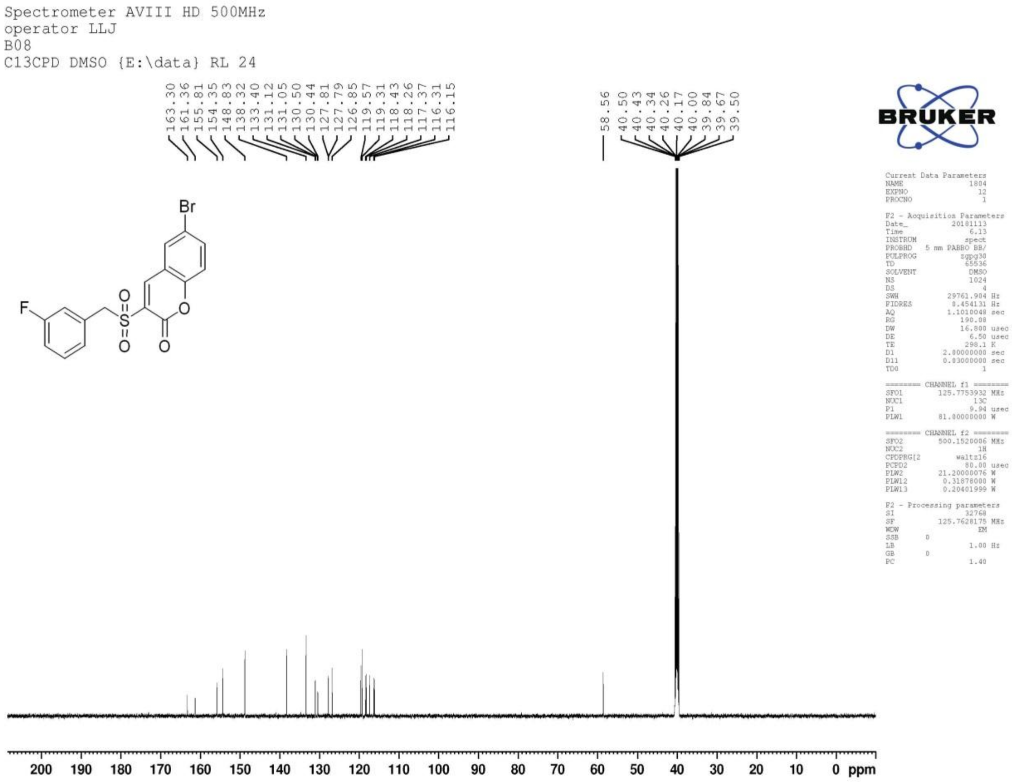

^{1}H-NMR of Compound **5l**:

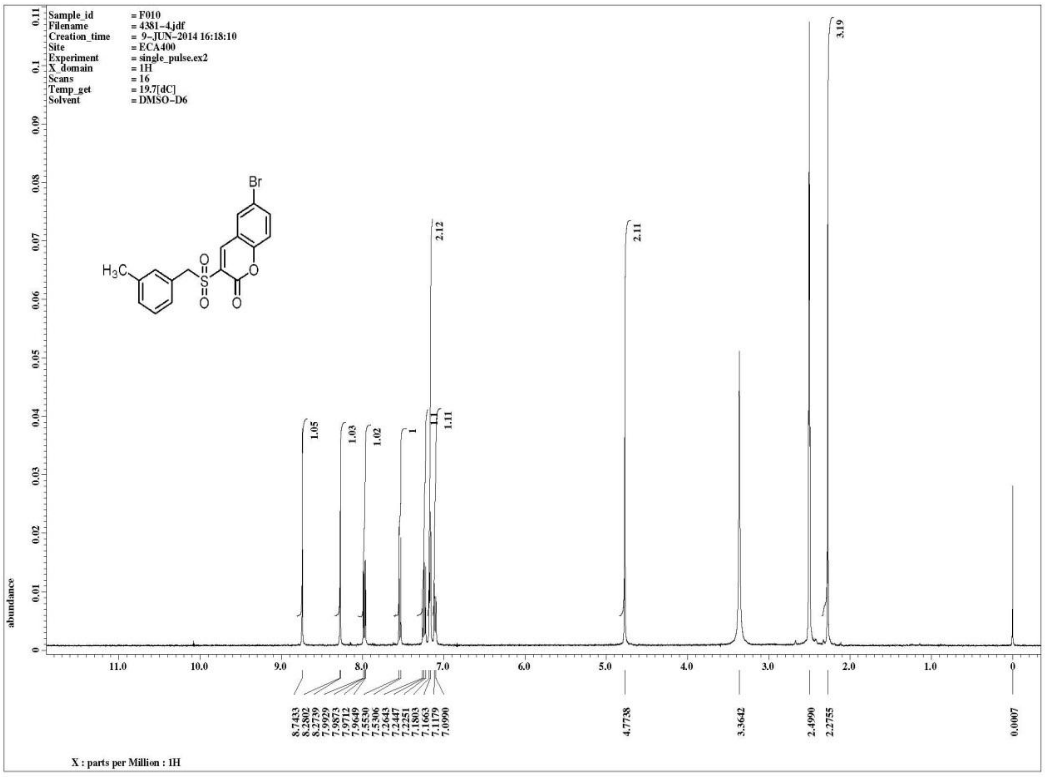

^{13}C-NMR of Compound **5l**:

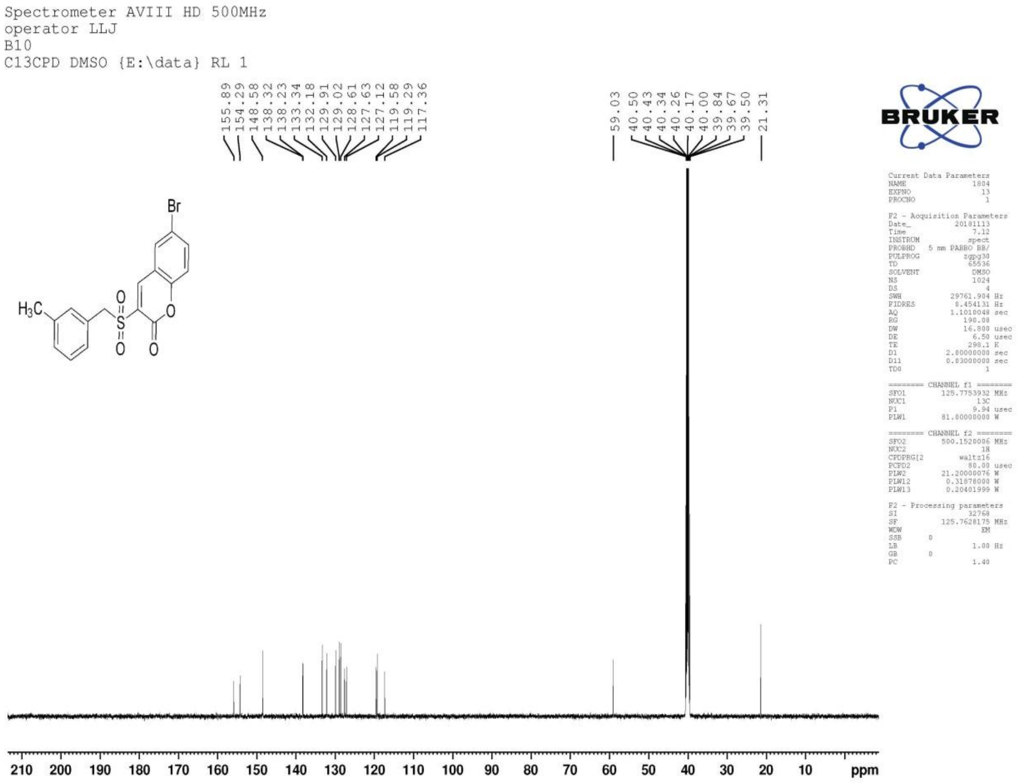

^{1}H-NMR of Compound **5m**:

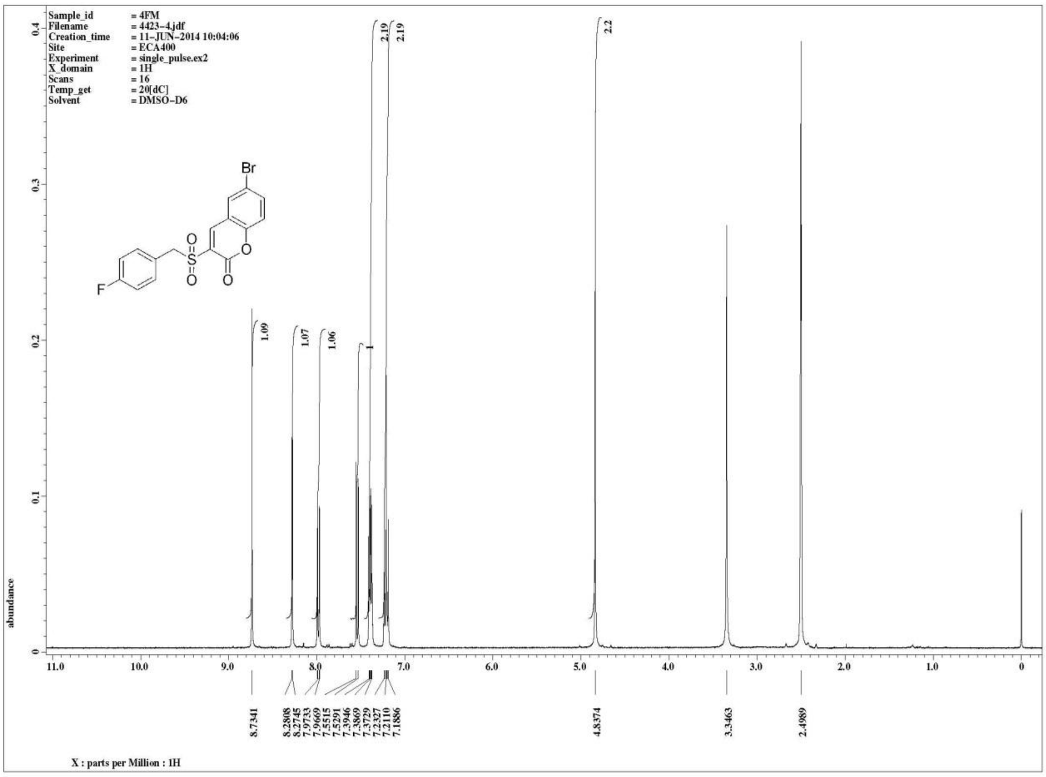

^{13}C-NMR of Compound **5m**:

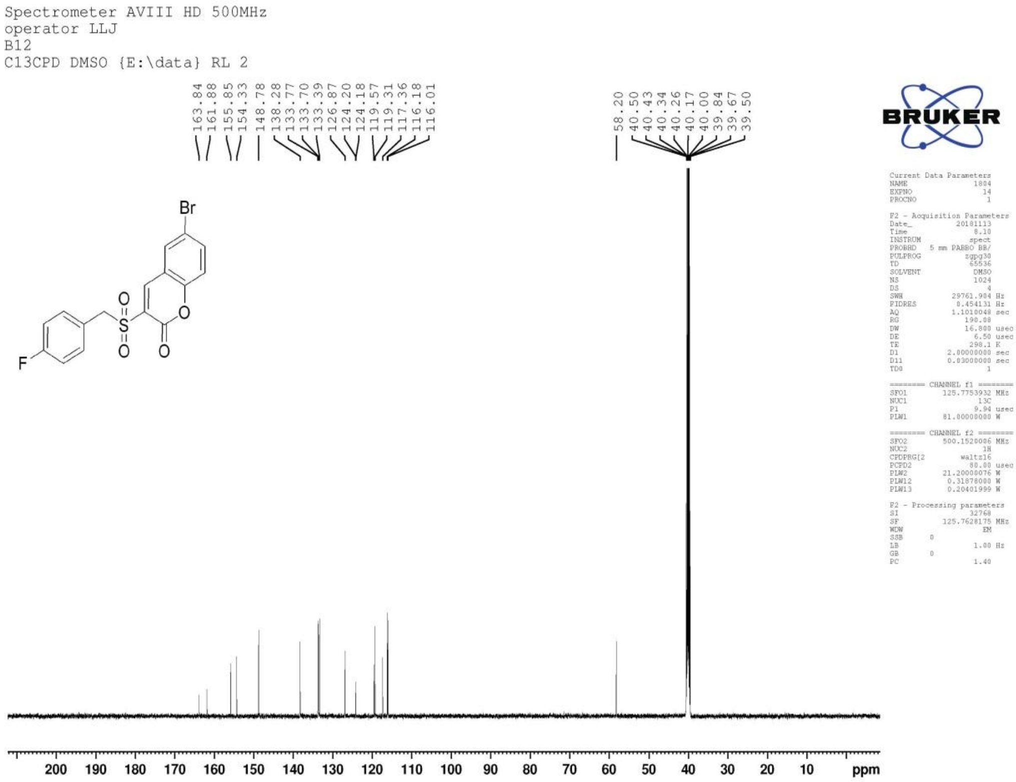

^{1}H-NMR of Compound **5n**:

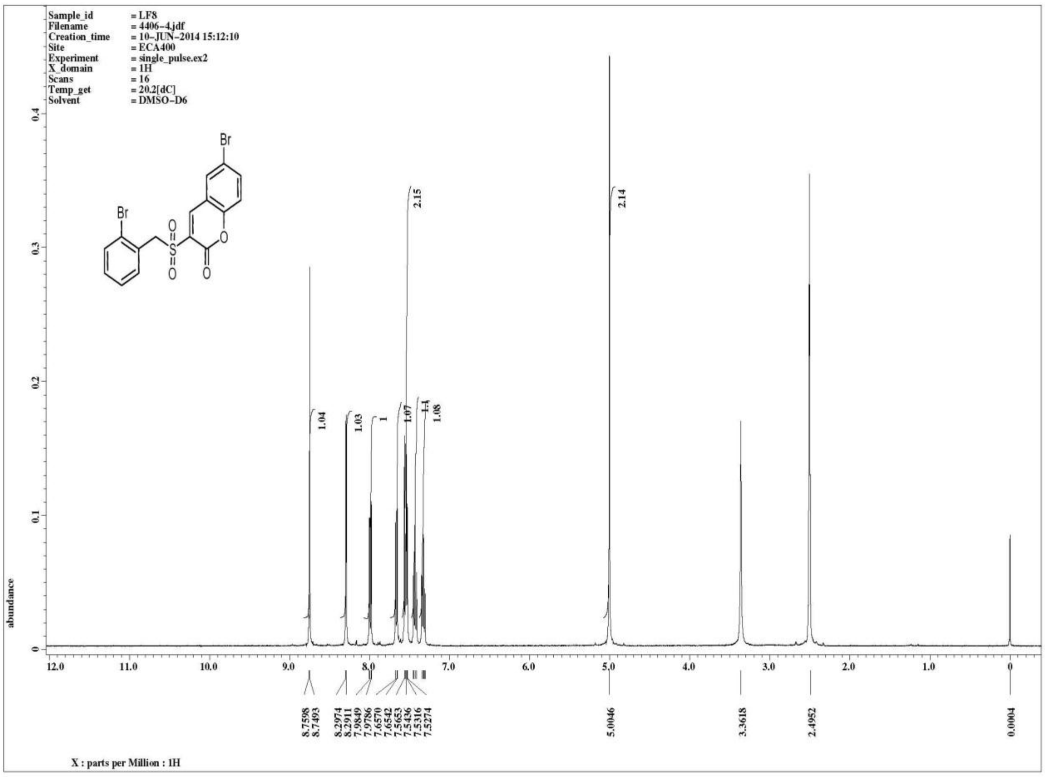

^{13}C-NMR of Compound **5n**:

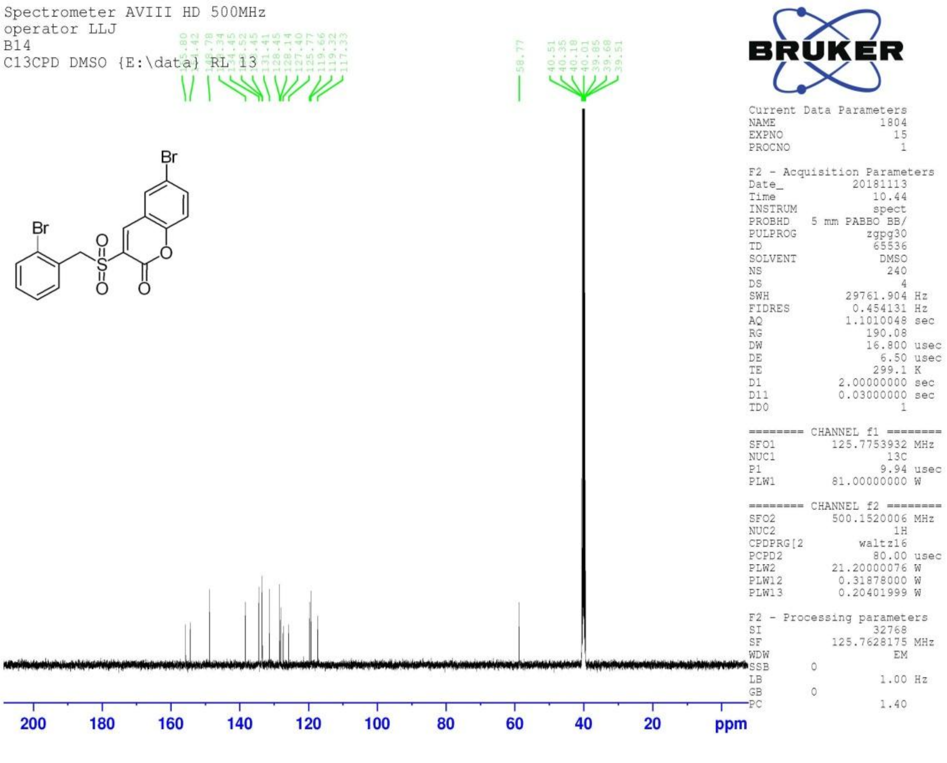

^{1}H-NMR of Compound **5o**:

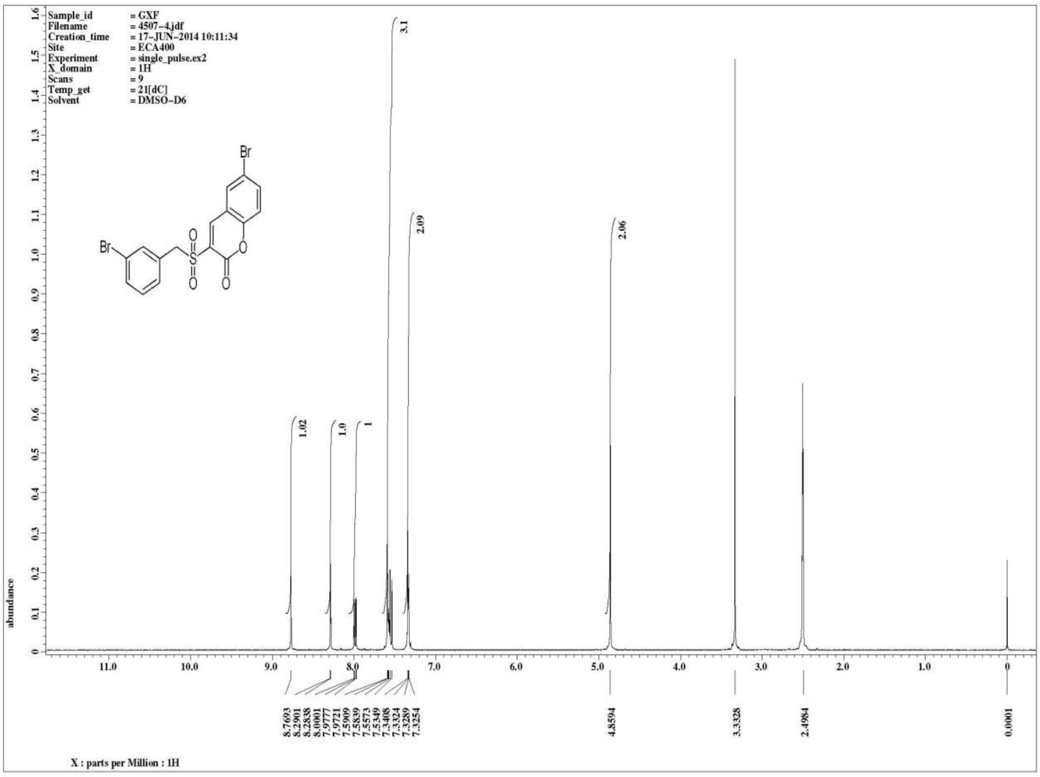

^{13}C-NMR of Compound **5o**:

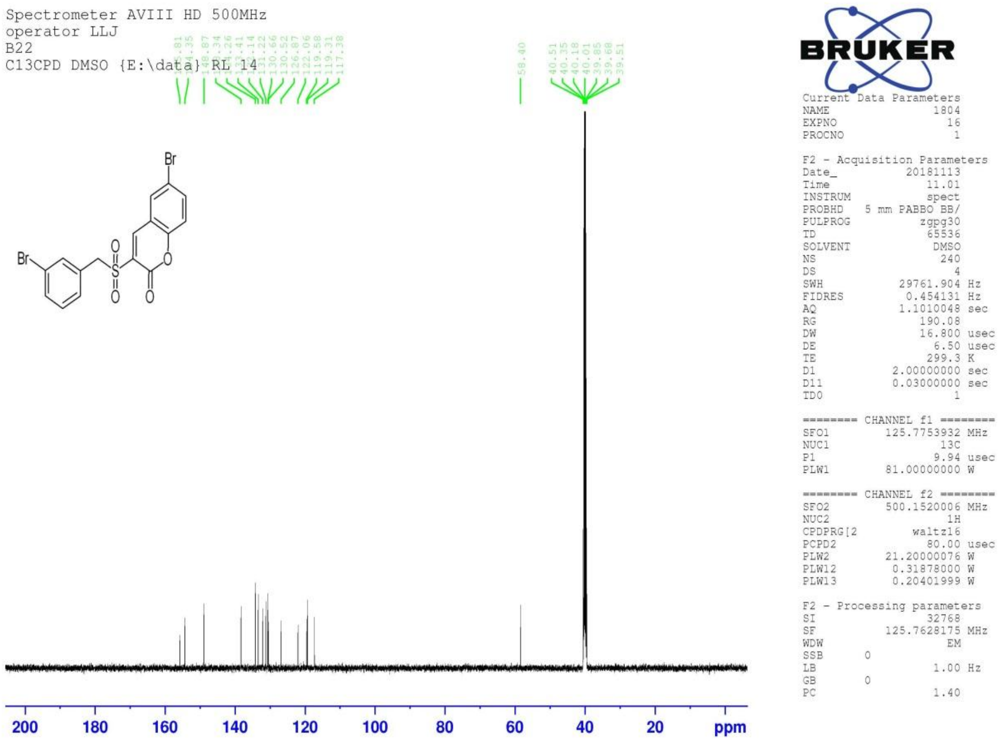

Supplement: Supplementary file 1 [file molecules-24-04034-s001.pdf]
